# What Pediatricians Need to Know About the CDC Guideline on the Diagnosis and Management of mTBI

**DOI:** 10.3389/fped.2018.00249

**Published:** 2018-09-12

**Authors:** Meeryo C. Choe, Andrew J. Gregory, Tamara M. Haegerich

**Affiliations:** ^1^UCLA Steve Tisch BrainSPORT Program, Division of Pediatric Neurology, Department of Pediatrics, UCLA Mattel Children's Hospital, David Geffen School of Medicine, Los Angeles, CA, United States; ^2^Vanderbilt Sports Concussion Center, Department of Orthopedics and Rehabilitation Sports Medicine Center, Vanderbilt University School of Medicine, Nashville, TN, United States; ^3^Centers for Disease Control and Prevention, National Center for Injury Prevention and Control, Atlanta, GA, United States

**Keywords:** guideline, concussion, traumatic brain injury, concussion diagnosis, concussion management

## Abstract

Pediatric traumatic brain injury (TBI) is a growing health concern, with over half a million TBI-related emergency department (ED) visits annually. However, this is likely an underestimate of the true incidence, with many children presenting to their pediatrician. The Centers for Disease Control and Prevention (CDC) published a guideline on the diagnosis and management of pediatric mild traumatic brain injury (mTBI). We outline key points and a decision checklist for pediatricians based on this evidence-based guideline.

## Background

Pediatric traumatic brain injury (TBI) is a growing public health concern. In 2013, there were an estimated 641,935 TBI-related emergency department (ED) visits, 17,930 TBI-related hospitalizations, and 1,484 TBI-related deaths among children aged 0–14 years in the United States (1). These data underestimate the true burden as the majority of pediatric patients with a mild traumatic brain injury (mTBI), which includes concussion, may seek care from an outpatient clinic or not seek care at all. In a study of point of entry within the Children's Hospital of Philadelphia system, Arbogast et al. found that 82% of patients had their first concussion-related visit within primary care, with only 12% entering care through the ED (2). With legislation in all 50 states requiring evaluation or diagnosis prior to return to play after a sports-related mTBI in children and adolescents, pediatricians may see a growing number of children in their offices with such injuries. These statistics point to the necessity of evidence-based recommendations for pediatricians. While mTBI guidelines are available for use in the adult population, comprehensive evidence-based guidelines for the diagnosis and management of pediatric mTBI have not been developed in the United States (3).

## The CDC guideline on diagnosis and management of mTBI among children

The Centers for Disease Control and Prevention (CDC) released evidence-based clinical recommendations on the diagnosis and management of pediatric mTBI. The *CDC Guideline on the Diagnosis and Management of Mild Traumatic Brain Injury Among Children* offers practical recommendations for pediatricians (and other specialties) to improve outcomes of children 18 years of age and younger. CDC used a broad clinical and functional definition of pediatric mTBI; that is, studies informing the Guideline included children described in the literature as having an mTBI by historical definitions, encompassing Glasgow Coma Scale scores of 13–15 with or without the complication of intracranial injury on neuroimaging, and regardless of the potential need for a hospital admission and/or neurological intervention (3). The Guideline provides: (1) diagnostic recommendations regarding the utility of head imaging, symptom scales, cognitive testing, and biomarkers; (2) prognostic recommendations that focus on counseling, assessing history, and risk, tools to track recovery, and interventions for patients with poor prognosis; and (3) management and treatment recommendations that focus on education, counseling, emotional support, return to activity and school, and management of specific symptoms.

The Guideline was developed through a rigorous federal advisory scientific process with expert input from a wide variety of perspectives (clinical providers, researchers, patients) and specialties (pediatrics, neurology, emergency medicine, sports medicine). Clinical questions were identified, and a systematic review of the literature was conducted. Evidence was rated using a modified Grading of Recommendation Assessment Development and Evaluation (GRADE) approach and used to inform recommendations on the diagnosis, prognosis, and management of mTBI in the context of scientific principles, related evidence, and expert inference.

**Figure 1:**
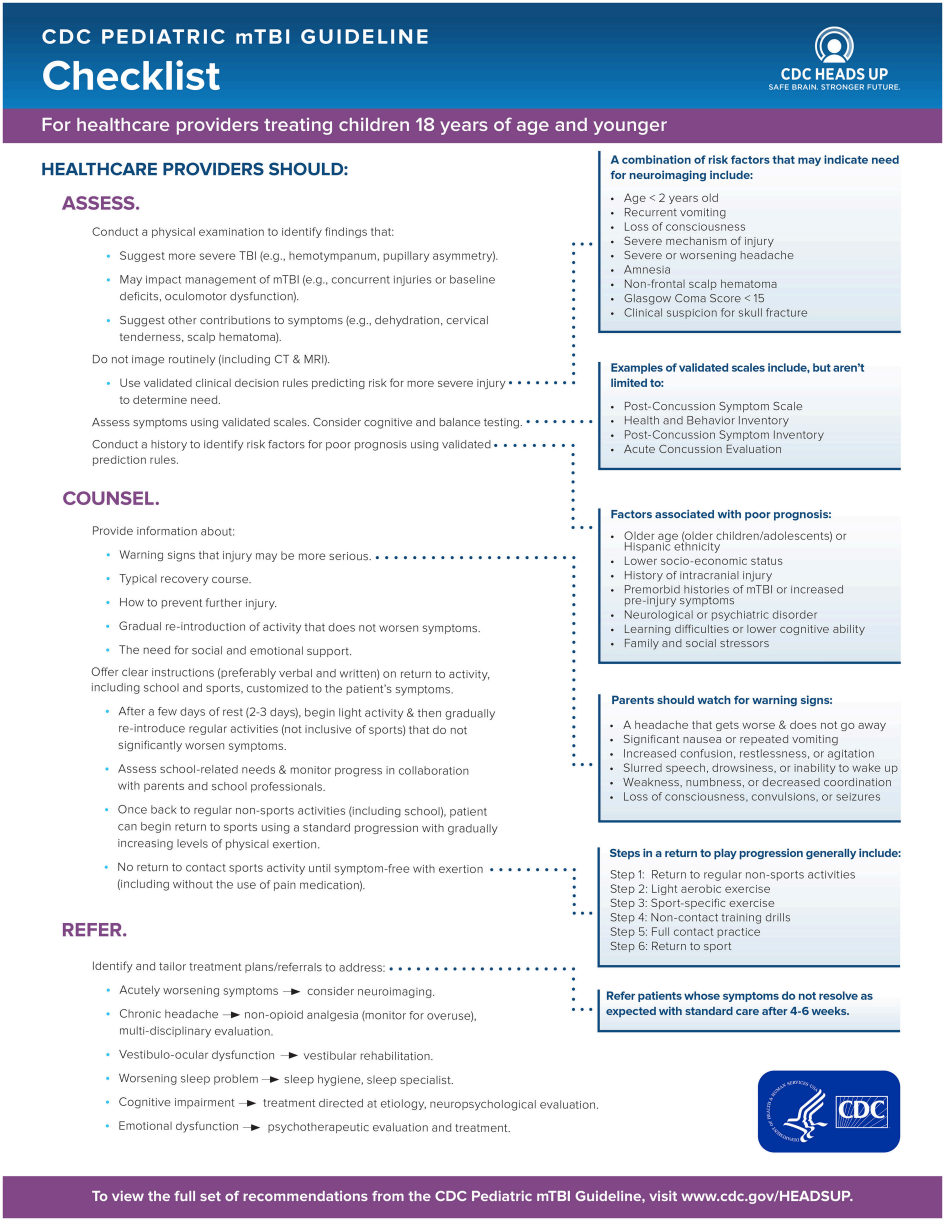
Checklist for diagnosing and managing mild traumatic brain injury (mTBI) in children.

The Guideline puts forth 19 sets of recommendations organized by clinical focus area. Recommendations are categorized by obligation (must, should, may) addressing the following topics: imaging, symptom scales, cognitive testing, the use of standardized testing for diagnosis, relevant history, and risk factor assessment, monitoring after injury, counseling regarding prognosis, and referral for further treatment. In this Perspective, we highlight recommendations from the CDC Guideline that are deemed to be the most relevant for the practicing pediatrician, prioritized by CDC for dissemination. In particular, we emphasize recommendations that apply to longer-term symptom management and return to activity, such as to school and sports, for pediatricians who not only see children at an initial visit for diagnosis, but also engage in regular follow-up—which can be uniquely different from other providers, such as emergency medicine practitioners. See Figure 1 for a checklist for providers on steps to take in diagnosing and managing mTBI in children.

## Key recommendations for pediatricians

### Diagnosis and management

Many tools to assess mTBI symptoms, prognosis, and recovery exist, but few are validated in the pediatric population, and none should be used in isolation. Initial evaluation consists of a combination of thorough history including screening for known risk factors for poor prognosis and the use of validated and age-appropriate tools for diagnosis. CDC recommends that this include a validated, age-appropriate symptom rating scale as a component [e.g., Post-Concussion Symptom Scale from ImPACT (4), Health Behavior Inventory (5), Post-Concussion Symptom Inventory (6); see Figure 1]. The use of computerized cognitive testing (e.g., ImPACT) may be helpful as an adjunctive test for diagnosis, but should not be used in isolation. A combination of tools may be useful in assessing recovery including validated symptom scales, cognitive testing, and balance testing. Going beyond the specific tools recommended in the Guideline, the National Academies report on sports-related concussions in youth provides additional information about tools that are available for use in the sport context (e.g., the Sport Concussion Assessment Tool for the sideline evaluation of athletes) (7). Clinicians can base selection of symptom rating scales and tools on the age of the child or adolescent being assessed, as well as the context of the injury. For example, Gioia et al. provide a review of clinical tools and symptom scales that are particularly useful for assessing student-athletes in the sports context (8).

Once the diagnosis of mTBI is made, pediatricians should counsel the child and his/her family that the majority (70–80%) of children recover within 1–3 months without difficulty. Education for the child and his/her family should include warning signs of more serious injury, how to prevent further injury (e.g., gradual return to activities), how to monitor symptoms, management of recovery through rest, and instructions on returning to cognitive and physical activity/recreation. By providing education and reassurance, one may avoid prolonged symptoms. Figure 1 provides further guidance on information to provide patients and their families (see *Counsel* step).

Pediatricians should recommend a brief period of rest (2–3 days) from physical and cognitive activities, which may be followed by symptom-limited activity. Too much rest beyond this period may worsen a child's symptoms and prolong recovery. Cognitive activity should be reintroduced gradually followed by physical activity, both of which should follow a gradual return to learn or play protocol with close monitoring for exacerbation of symptoms. A child may return to full pre-injury activity when symptom-free at rest and with stepwise progression of physical exertion. Active recovery may decrease the presence of post-concussive symptoms in those children who have prolonged symptoms (3). Symptomatic treatment for headache may include non-opioid analgesics such as acetaminophen or ibuprofen.

### When to refer for imaging

CDC recommends that pediatricians should not routinely image a child presenting with suspected mTBI (see *Assess* step in Figure 1). Rather, clinicians should use validated clinical decision rules to determine the need for imaging based on factors that increase risk for injury (e.g., age < 2 years, vomiting, loss of consciousness, severe or worsening headache, amnesia), and counsel the patient and family regarding the risks associated with imaging. However, pediatricians should refer a child for imaging if the presenting symptom is severe headache with additional risk factors or worsening headache for imaging to assess for possible intracranial injury. There is moderate risk for a clinically important intracranial injury (e.g., hemorrhage) when a child presents with a mild injury (Glasgow Coma Score 13–15) and a severe or worsening headache. Validated clinical decision rules can help to identify those children at higher risk for a clinically important intracranial injury, and who should be referred for head CT (see Figure 1 for factors that assist in determining need for imaging) (9).

### When to refer for further treatment

If symptoms do not resolve within 4–6 weeks, further assessment and intervention may be needed. There may be risk factors present for prolonged symptoms; these can be identified when obtaining the patient's past medical and family history. Children who have a history of TBI, neurological, or psychiatric disorder, learning difficulties, or family or social stressors may be at risk for delayed recovery. Pediatricians may use validated prediction rules that combine information about such risk factors to identify children at highest probability for persistent symptoms (see Figure 1 for factors associated with delayed recovery). These children and their families may be counseled accordingly, and may warrant earlier referral to a specialized clinic for close follow-up.

The majority of children will return to baseline within 1–3 months. Persistent symptoms may require specialized intervention. Children with persistent vestibulo-oculomotor dysfunction may benefit from vestibular rehabilitation. Persistent cognitive impairment should also be treated with specific consideration of the presumed etiology. Formal neuropsychological evaluation may help in determining the etiology and appropriate treatment (see *Refer* step in Figure 1).

## Conclusion

CDC offers tools to assist providers in implementing the Guideline recommendations in clinical practice, as well as in assisting children in returning to activity/play and school. The CDC HEADS UP materials for healthcare providers (www.cdc.gov/headsup/providers) includes free online training with continuing education credits available, as well as the provider checklist, diagnostic and assessment tools, discharge instructions, and guidance on return to play and return to learn protocols (10). Integration of recommendations using prompts and alerts within the electronic health record could also assist pediatricians with implementation at the point of care, particularly with return to learn and return to play recommendations (11).

## Author contributions

MC and TH drafted the initial manuscript, and reviewed and revised the manuscript. AG reviewed and revised the manuscript. All authors approved the final manuscript as submitted and agree to be accountable for all aspects of the work.

### Conflict of interest statement

The authors declare that the research was conducted in the absence of any commercial or financial relationships that could be construed as a potential conflict of interest.

## Abbreviations

CDC: Centers for Disease Control and Prevention
TBI: traumatic brain injury
mTBI: mild traumatic brain injury
ED: emergency department
CT: computed tomography.
